# 1-(3,5-Dimethyl-1*H*-pyrazol-1-yl)-3-phenyl­isoquinoline

**DOI:** 10.1107/S1600536809024842

**Published:** 2009-07-08

**Authors:** P. Manivel, Venkatesha R. Hathwar, T. Maiyalagan, V. Krishnakumar, F. Nawaz Khan

**Affiliations:** a Chemistry Division, School of Science and Humanities, VIT University, Vellore 632 014, Tamil Nadu, India; b Solid State and Structural Chemistry Unit, Indian Institute of Science, Bangalore 560 012, Karnataka, India

## Abstract

The mol­ecular conformation of the title compound, C_20_H_17_N_3_, is stabilized by an intramolecular C—H⋯N inter­action. The crystal structure shows inter­molecular C—H⋯π inter­actions. The dihedral angle between the isoquinoline unit and the phenyl ring is 11.42 (1)° whereas the isoquinoline unit and the pendent dimethyl pryrazole unit form a dihedral angle of 50.1 (4)°. Furthermore, the angle between the mean plane of the phenyl ring and the dimethyl pyrazole unit is 47.3 (6)°.

## Related literature

For general background to isoquinolines, see: Kametani *et al.* (1968 ▶); Broadhurst *et al.* (2001 ▶); Chao *et al.* (1999 ▶); Choudhury *et al.* (2002 ▶, 2006 ▶); Hathwar *et al.* (2008 ▶); Elguero *et al.* 2002 ▶).
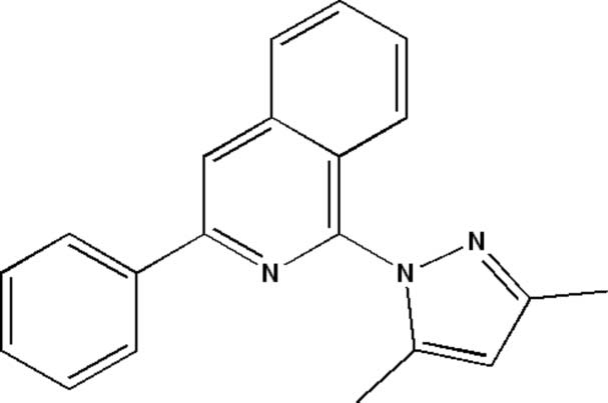


## Experimental

### 

#### Crystal data

C_20_H_17_N_3_
*M* *_r_* = 299.37
Orthorhombic, 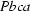

*a* = 18.3294 (14) Å
*b* = 8.3139 (7) Å
*c* = 21.6532 (17) Å
*V* = 3299.7 (5) Å^3^
*Z* = 8
Mo *K*α radiation
μ = 0.07 mm^−1^
*T* = 290 K
0.18 × 0.11 × 0.07 mm

#### Data collection

Bruker SMART CCD area-detector diffractometer
Absorption correction: multi-scan (*SADABS*; Sheldrick, 1996 ▶) *T*_min_ = 0.949, *T*_max_ = 0.995
22808 measured reflections
3065 independent reflections
2608 reflections with *I* > 2σ(*I*)
*R*_int_ = 0.027

#### Refinement

*R*[*F* ^2^ > 2σ(*F* ^2^)] = 0.052
*wR*(*F* ^2^) = 0.129
*S* = 1.14
3065 reflections
276 parameters
All H-atom parameters refined
Δρ_max_ = 0.15 e Å^−3^
Δρ_min_ = −0.15 e Å^−3^

### 

Data collection: *SMART* (Bruker, 2004 ▶); cell refinement: *SAINT* (Bruker, 2004 ▶); data reduction: *SAINT*; program(s) used to solve structure: *SHELXS97* (Sheldrick, 2008 ▶); program(s) used to refine structure: *SHELXL97* (Sheldrick, 2008 ▶); molecular graphics: *CAMERON* (Watkin *et al.*, 1993 ▶); software used to prepare material for publication: *PLATON* (Spek, 2009 ▶).

## Supplementary Material

Crystal structure: contains datablocks global, I. DOI: 10.1107/S1600536809024842/bt2974sup1.cif

Structure factors: contains datablocks I. DOI: 10.1107/S1600536809024842/bt2974Isup2.hkl

Additional supplementary materials:  crystallographic information; 3D view; checkCIF report

## Figures and Tables

**Table 1:** Hydrogen-bond geometry (Å, °)

*D*—H⋯*A*	*D*—H	H⋯*A*	*D*⋯*A*	*D*—H⋯*A*
C7—H7⋯N3	0.94 (2)	2.452 (17)	3.001 (2)	117.4 (13)
C4—H4⋯*Cg*2^i^	0.91 (2)	2.645 (17)	3.325 (2)	131.4 (13)

Symmetry code: (i) 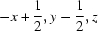. *Cg*2 is the centroid of the N1,C1–C3,C8,C9 ring.

**Fig. 1.**
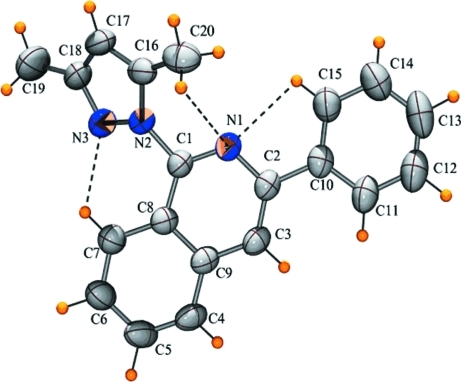
ORTEP diagram of the asymmetric unit of (I) with 50% probability displacement ellipsoids.

**Fig. 2.**
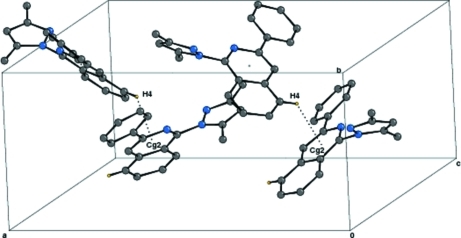
A packing excerpt from the crystal with dotted lines indicating intermolecular C—H···π hydrogen bonds. H atoms not involved in the interactions are omitted for clarity.

## supplementary crystallographic information

### Comment

Isoquinolines are an integral part of many naturally occurring fused heterocycles and find applications in synthetic and pharmaceutical chemistry (Kametani *et al.*, 1968). 3-substituted isoquinolines are of potent use in medicine, (Chao *et al.*, 1999) and in general, hydrazine derivatives can be used as medicaments (Broadhurst *et al.*, 2001). Choudhury *et al.* (2002, 2006) reported crystal structures of substituted isoquinolines while Hathwar  *et al.* (2008) report  the crystal structure of an isoquinolinyl diselenide. Similarly, compounds containing the pyrazole motif are being developed in a wide range of therapeutic areas including *CNS*, metabolic diseases and endocrine functions, and oncology (Elguero *et al.*, 2002). A number of pyrazole-containing compounds have been successfully commercialized, such as the blockbuster drugs Viagra, Celebrex, and Acomplia. In view of the diverse applications of this class of compounds, we report here the crystal structure of isoquinoline pyrazole, namely 1-(2,5-dimethyl-1*H*-pyrrol-1-yl)-3-phenylisoquinoline.

Although there are no intermolecular C—H···N hydrogen bonds, the molecules are linked   by C—H···π interactions.

In the absence of strong hydrogen-bond donors in (I), the crystal packing is controlled by the involvement of weak C—H..pi intermolecular interactions.

### Experimental

The 3-phenylisoquinolinehydrazine, and the 1, 3-diketones namely acetylacetone, were taken in ethanol (1:1 ratio) and refluxed under nitrogen overnight. Then the reaction mass was quenched with water, extracted with ethylacetate, washed, dried, concentrated and purified by column chromatography to get titlted compound, (I). Single crystals of the title compound were obtained *via* recrystalization from a dichloromethane solution

### Refinement

All the H atoms in (I) were positioned geometrically and refined using a riding model with C—H bond lenghts of 0.93 Å and 0.97 Å for aromatic and for methylene H atoms respectively and *U*_iso_(H) = 1.2*U*_eq_(C) for all carbon bound H atoms.

### Crystal data

**Table:**

C_20_H_17_N_3_	*F*(000) = 1264
*M**_r_* = 299.37	*D*_x_ = 1.205 Mg m^−^^3^
Orthorhombic, *P**b**c**a*	Mo *K*α radiation, λ = 0.71073 Å
Hall symbol: -P 2ac 2ab	Cell parameters from 1248 reflections
*a* = 18.3294 (14) Å	θ = 2.2–27.2°
*b* = 8.3139 (7) Å	µ = 0.07 mm^−^^1^
*c* = 21.6532 (17) Å	*T* = 290 K
*V* = 3299.7 (5) Å^3^	Block, colorless
*Z* = 8	0.18 × 0.11 × 0.07 mm

### Data collection

**Table:**

Bruker SMART CCD area-detector diffractometer	3065 independent reflections
Radiation source: fine-focus sealed tube	2608 reflections with *I* > 2σ(*I*)
graphite	*R*_int_ = 0.027
φ and ω scans	θ_max_ = 25.5°, θ_min_ = 1.9°
Absorption correction: multi-scan (*SADABS*; Sheldrick, 1996)	*h* = −22→20
*T*_min_ = 0.949, *T*_max_ = 0.995	*k* = −10→10
22808 measured reflections	*l* = −26→24

### Refinement

**Table:**

Refinement on *F*^2^	Primary atom site location: structure-invariant direct methods
Least-squares matrix: full	Secondary atom site location: difference Fourier map
*R*[*F*^2^ > 2σ(*F*^2^)] = 0.052	Hydrogen site location: inferred from neighbouring sites
*wR*(*F*^2^) = 0.129	All H-atom parameters refined
*S* = 1.14	*w* = 1/[σ^2^(*F*_o_^2^) + (0.0517*P*)^2^ + 0.6765*P*] where *P* = (*F*_o_^2^ + 2*F*_c_^2^)/3
3065 reflections	(Δ/σ)_max_ < 0.001
276 parameters	Δρ_max_ = 0.14 e Å^−^^3^
0 restraints	Δρ_min_ = −0.15 e Å^−^^3^

### Special details

**Table:**

Geometry. All e.s.d.'s (except the e.s.d. in the dihedral angle between two l.s. planes) are estimated using the full covariance matrix. The cell e.s.d.'s are taken into account individually in the estimation of e.s.d.'s in distances, angles and torsion angles; correlations between e.s.d.'s in cell parameters are only used when they are defined by crystal symmetry. An approximate (isotropic) treatment of cell e.s.d.'s is used for estimating e.s.d.'s involving l.s. planes.
Refinement. Refinement of *F*^2^ against ALL reflections. The weighted *R*-factor *wR* and goodness of fit *S* are based on *F*^2^, conventional *R*-factors *R* are based on *F*, with *F* set to zero for negative *F*^2^. The threshold expression of *F*^2^ > σ(*F*^2^) is used only for calculating *R*-factors(gt) *etc*. and is not relevant to the choice of reflections for refinement. *R*-factors based on *F*^2^ are statistically about twice as large as those based on *F*, and *R*- factors based on ALL data will be even larger.

### Fractional atomic coordinates and isotropic or equivalent isotropic displacement parameters (Å^2^)

**Table:**

	*x*	*y*	*z*	*U*_iso_*/*U*_eq_	
N1	0.09848 (7)	0.53396 (16)	0.26708 (6)	0.0490 (4)	
N2	0.02198 (7)	0.52682 (18)	0.18301 (6)	0.0538 (4)	
C1	0.08768 (9)	0.47659 (19)	0.21175 (7)	0.0470 (4)	
C8	0.13594 (9)	0.37101 (19)	0.18015 (7)	0.0477 (4)	
C3	0.21303 (10)	0.3989 (2)	0.27066 (8)	0.0525 (4)	
C9	0.20196 (9)	0.3351 (2)	0.21115 (8)	0.0491 (4)	
C2	0.16088 (9)	0.4924 (2)	0.29813 (8)	0.0485 (4)	
C10	0.16626 (9)	0.5586 (2)	0.36145 (8)	0.0533 (4)	
C4	0.25285 (11)	0.2347 (2)	0.18064 (9)	0.0611 (5)	
C16	−0.04605 (9)	0.5383 (2)	0.20820 (9)	0.0555 (5)	
C7	0.12169 (11)	0.3011 (2)	0.12208 (8)	0.0597 (5)	
N3	0.02521 (8)	0.5925 (2)	0.12480 (7)	0.0677 (5)	
C20	−0.06494 (13)	0.4719 (3)	0.26970 (12)	0.0742 (6)	
C5	0.23812 (12)	0.1701 (3)	0.12503 (9)	0.0705 (6)	
C11	0.22045 (12)	0.5099 (3)	0.40250 (9)	0.0673 (6)	
C15	0.11577 (12)	0.6709 (3)	0.38203 (9)	0.0698 (6)	
C6	0.17167 (12)	0.2015 (3)	0.09549 (10)	0.0699 (6)	
C12	0.22252 (14)	0.5691 (3)	0.46184 (10)	0.0819 (7)	
C18	−0.04247 (12)	0.6423 (3)	0.11454 (9)	0.0723 (6)	
C13	0.17137 (16)	0.6770 (3)	0.48194 (11)	0.0876 (8)	
C17	−0.08729 (12)	0.6106 (3)	0.16449 (10)	0.0700 (6)	
C14	0.11810 (15)	0.7294 (3)	0.44162 (11)	0.0855 (7)	
C19	−0.06113 (15)	0.7207 (4)	0.05432 (11)	0.1147 (10)	
H19A	−0.0312	0.8144	0.0487	0.172*	
H19B	−0.1116	0.7517	0.0545	0.172*	
H19C	−0.0526	0.6464	0.0212	0.172*	
H3	0.2583 (10)	0.378 (2)	0.2919 (8)	0.064 (5)*	
H7	0.0783 (10)	0.330 (2)	0.1015 (8)	0.070 (6)*	
H4	0.2958 (10)	0.212 (2)	0.2010 (9)	0.065 (5)*	
H11	0.2580 (13)	0.431 (3)	0.3893 (10)	0.090 (7)*	
H5	0.2750 (11)	0.100 (3)	0.1032 (9)	0.080 (6)*	
H15	0.0784 (11)	0.712 (3)	0.3537 (9)	0.079 (6)*	
H20C	−0.1161 (15)	0.482 (3)	0.2777 (10)	0.106 (8)*	
H17	−0.1358 (12)	0.633 (2)	0.1678 (9)	0.073 (6)*	
H20B	−0.0531 (14)	0.356 (4)	0.2713 (12)	0.128 (10)*	
H14	0.0827 (11)	0.809 (3)	0.4543 (10)	0.083 (7)*	
H12	0.2612 (13)	0.529 (3)	0.4907 (11)	0.104 (8)*	
H20A	−0.0422 (13)	0.531 (3)	0.3038 (11)	0.098 (8)*	
H6	0.1605 (11)	0.153 (3)	0.0533 (11)	0.090 (7)*	
H13	0.1701 (12)	0.718 (3)	0.5233 (12)	0.099 (8)*	

### Atomic displacement parameters (Å^2^)

**Table:**

	*U*^11^	*U*^22^	*U*^33^	*U*^12^	*U*^13^	*U*^23^
N1	0.0454 (8)	0.0533 (8)	0.0483 (8)	−0.0005 (6)	−0.0008 (6)	0.0040 (6)
N2	0.0449 (8)	0.0652 (9)	0.0513 (8)	0.0098 (7)	−0.0043 (6)	−0.0032 (7)
C1	0.0424 (9)	0.0510 (9)	0.0474 (9)	0.0001 (7)	−0.0008 (7)	0.0054 (7)
C8	0.0459 (9)	0.0484 (9)	0.0489 (9)	0.0017 (7)	0.0051 (7)	0.0088 (7)
C3	0.0449 (9)	0.0588 (10)	0.0540 (10)	0.0024 (8)	−0.0011 (8)	0.0162 (8)
C9	0.0457 (9)	0.0503 (9)	0.0512 (10)	0.0033 (7)	0.0052 (7)	0.0168 (7)
C2	0.0468 (9)	0.0506 (9)	0.0480 (9)	−0.0056 (7)	−0.0009 (7)	0.0118 (7)
C10	0.0545 (10)	0.0578 (10)	0.0478 (10)	−0.0113 (8)	−0.0001 (8)	0.0106 (8)
C4	0.0567 (11)	0.0659 (11)	0.0607 (12)	0.0191 (9)	0.0073 (9)	0.0213 (9)
C16	0.0437 (9)	0.0549 (10)	0.0679 (12)	0.0022 (8)	−0.0008 (8)	−0.0161 (9)
C7	0.0632 (12)	0.0634 (11)	0.0524 (11)	0.0101 (9)	−0.0005 (9)	0.0026 (9)
N3	0.0647 (10)	0.0857 (12)	0.0528 (9)	0.0237 (9)	−0.0041 (7)	0.0024 (8)
C20	0.0531 (13)	0.0783 (16)	0.0912 (17)	−0.0012 (11)	0.0162 (12)	−0.0037 (13)
C5	0.0844 (15)	0.0722 (13)	0.0550 (12)	0.0320 (11)	0.0164 (11)	0.0138 (10)
C11	0.0665 (13)	0.0829 (14)	0.0526 (12)	−0.0092 (11)	−0.0073 (9)	0.0133 (10)
C15	0.0766 (14)	0.0766 (13)	0.0563 (12)	−0.0002 (11)	−0.0052 (10)	−0.0015 (10)
C6	0.0863 (15)	0.0710 (13)	0.0524 (11)	0.0241 (11)	0.0046 (10)	0.0018 (10)
C12	0.0833 (16)	0.1038 (18)	0.0586 (13)	−0.0167 (14)	−0.0144 (12)	0.0141 (13)
C18	0.0707 (13)	0.0816 (14)	0.0647 (12)	0.0302 (11)	−0.0169 (10)	−0.0101 (10)
C13	0.114 (2)	0.0989 (18)	0.0499 (13)	−0.0335 (16)	−0.0056 (13)	−0.0015 (12)
C17	0.0485 (11)	0.0761 (13)	0.0853 (15)	0.0197 (10)	−0.0127 (10)	−0.0192 (11)
C14	0.1017 (18)	0.0896 (16)	0.0651 (14)	−0.0013 (15)	0.0035 (13)	−0.0127 (12)
C19	0.117 (2)	0.149 (3)	0.0785 (16)	0.065 (2)	−0.0211 (15)	0.0087 (16)

### Geometric parameters (Å, °)

**Table:**

N1—C1	1.305 (2)	C20—H20C	0.96 (3)
N1—C2	1.371 (2)	C20—H20B	0.99 (3)
N2—C16	1.364 (2)	C20—H20A	0.98 (3)
N2—N3	1.375 (2)	C5—C6	1.400 (3)
N2—C1	1.418 (2)	C5—H5	1.01 (2)
C1—C8	1.422 (2)	C11—C12	1.376 (3)
C8—C7	1.410 (2)	C11—H11	1.00 (2)
C8—C9	1.416 (2)	C15—C14	1.380 (3)
C3—C2	1.368 (2)	C15—H15	0.98 (2)
C3—C9	1.408 (2)	C6—H6	1.02 (2)
C3—H3	0.966 (18)	C12—C13	1.369 (4)
C9—C4	1.416 (2)	C12—H12	1.00 (3)
C2—C10	1.481 (2)	C18—C17	1.383 (3)
C10—C15	1.388 (3)	C18—C19	1.498 (3)
C10—C11	1.393 (3)	C13—C14	1.380 (3)
C4—C5	1.346 (3)	C13—H13	0.96 (2)
C4—H4	0.922 (18)	C17—H17	0.91 (2)
C16—C17	1.353 (3)	C14—H14	0.97 (2)
C16—C20	1.482 (3)	C19—H19A	0.9600
C7—C6	1.362 (3)	C19—H19B	0.9600
C7—H7	0.943 (19)	C19—H19C	0.9600
N3—C18	1.326 (2)		
			
C1—N1—C2	119.00 (14)	H20C—C20—H20A	103.7 (19)
C16—N2—N3	112.22 (14)	H20B—C20—H20A	112 (2)
C16—N2—C1	128.39 (15)	C4—C5—C6	120.56 (19)
N3—N2—C1	118.85 (13)	C4—C5—H5	121.0 (11)
N1—C1—N2	115.09 (14)	C6—C5—H5	118.4 (11)
N1—C1—C8	124.98 (15)	C12—C11—C10	120.7 (2)
N2—C1—C8	119.92 (15)	C12—C11—H11	119.0 (13)
C7—C8—C9	119.62 (16)	C10—C11—H11	120.2 (13)
C7—C8—C1	124.66 (16)	C14—C15—C10	121.1 (2)
C9—C8—C1	115.72 (15)	C14—C15—H15	119.0 (12)
C2—C3—C9	120.75 (16)	C10—C15—H15	119.9 (12)
C2—C3—H3	119.8 (11)	C7—C6—C5	120.4 (2)
C9—C3—H3	119.4 (11)	C7—C6—H6	119.0 (12)
C3—C9—C4	123.67 (16)	C5—C6—H6	120.6 (12)
C3—C9—C8	118.52 (15)	C13—C12—C11	120.8 (2)
C4—C9—C8	117.80 (17)	C13—C12—H12	120.2 (14)
C3—C2—N1	120.85 (16)	C11—C12—H12	118.9 (15)
C3—C2—C10	124.57 (15)	N3—C18—C17	111.41 (18)
N1—C2—C10	114.57 (15)	N3—C18—C19	119.7 (2)
C15—C10—C11	117.79 (19)	C17—C18—C19	128.89 (19)
C15—C10—C2	120.19 (16)	C12—C13—C14	119.4 (2)
C11—C10—C2	122.00 (18)	C12—C13—H13	123.1 (14)
C5—C4—C9	121.39 (19)	C14—C13—H13	117.5 (14)
C5—C4—H4	121.1 (12)	C16—C17—C18	107.44 (18)
C9—C4—H4	117.5 (12)	C16—C17—H17	125.4 (13)
C17—C16—N2	105.19 (18)	C18—C17—H17	127.1 (13)
C17—C16—C20	131.6 (2)	C15—C14—C13	120.1 (3)
N2—C16—C20	123.14 (17)	C15—C14—H14	119.1 (13)
C6—C7—C8	120.20 (19)	C13—C14—H14	120.8 (13)
C6—C7—H7	121.4 (12)	C18—C19—H19A	109.5
C8—C7—H7	118.3 (12)	C18—C19—H19B	109.5
C18—N3—N2	103.72 (16)	H19A—C19—H19B	109.5
C16—C20—H20C	111.2 (14)	C18—C19—H19C	109.5
C16—C20—H20B	110.1 (16)	H19A—C19—H19C	109.5
H20C—C20—H20B	107 (2)	H19B—C19—H19C	109.5
C16—C20—H20A	112.9 (14)		
			
C2—N1—C1—N2	−179.60 (14)	N3—N2—C16—C17	1.0 (2)
C2—N1—C1—C8	0.5 (2)	C1—N2—C16—C17	172.43 (17)
C16—N2—C1—N1	−43.2 (2)	N3—N2—C16—C20	177.88 (18)
N3—N2—C1—N1	127.74 (16)	C1—N2—C16—C20	−10.7 (3)
C16—N2—C1—C8	136.70 (18)	C9—C8—C7—C6	−1.0 (3)
N3—N2—C1—C8	−52.4 (2)	C1—C8—C7—C6	179.82 (18)
N1—C1—C8—C7	175.60 (16)	C16—N2—N3—C18	−0.9 (2)
N2—C1—C8—C7	−4.3 (2)	C1—N2—N3—C18	−173.20 (16)
N1—C1—C8—C9	−3.6 (2)	C9—C4—C5—C6	0.4 (3)
N2—C1—C8—C9	176.55 (14)	C15—C10—C11—C12	1.4 (3)
C2—C3—C9—C4	−178.55 (16)	C2—C10—C11—C12	−177.56 (18)
C2—C3—C9—C8	0.6 (2)	C11—C10—C15—C14	−1.5 (3)
C7—C8—C9—C3	−176.34 (15)	C2—C10—C15—C14	177.43 (19)
C1—C8—C9—C3	2.9 (2)	C8—C7—C6—C5	−1.2 (3)
C7—C8—C9—C4	2.8 (2)	C4—C5—C6—C7	1.5 (3)
C1—C8—C9—C4	−177.96 (15)	C10—C11—C12—C13	0.1 (3)
C9—C3—C2—N1	−3.8 (2)	N2—N3—C18—C17	0.4 (2)
C9—C3—C2—C10	177.19 (15)	N2—N3—C18—C19	−179.4 (2)
C1—N1—C2—C3	3.3 (2)	C11—C12—C13—C14	−1.4 (4)
C1—N1—C2—C10	−177.61 (14)	N2—C16—C17—C18	−0.7 (2)
C3—C2—C10—C15	170.61 (17)	C20—C16—C17—C18	−177.2 (2)
N1—C2—C10—C15	−8.4 (2)	N3—C18—C17—C16	0.2 (3)
C3—C2—C10—C11	−10.5 (3)	C19—C18—C17—C16	180.0 (2)
N1—C2—C10—C11	170.48 (16)	C10—C15—C14—C13	0.2 (4)
C3—C9—C4—C5	176.59 (18)	C12—C13—C14—C15	1.3 (4)
C8—C9—C4—C5	−2.5 (3)		

### Hydrogen-bond geometry (Å, °)

**Table:**

*D*—H···*A*	*D*—H	H···*A*	*D*···*A*	*D*—H···*A*
C7—H7···N3	0.94 (2)	2.452 (17)	3.001 (2)	117.4 (13)
C4—H4···Cg2^i^	0.91 (2)	2.645 (17)	3.325 (2)	131.4 (13)

Symmetry codes: (i) −*x*+1/2, *y*−1/2, *z*.
